# Sensual, Erotic, and Sexual Behaviors of Women from the “Kink” Community

**DOI:** 10.1007/s10508-015-0524-2

**Published:** 2015-03-21

**Authors:** Jennifer Eve Rehor

**Affiliations:** Department of Sociology and Sexuality Studies, San Francisco State University, 1600 Holloway Ave., San Francisco, CA 94132 USA

**Keywords:** Kink, Kinky women, Female sexuality, Unconventional sexual behaviors, BDSM, Alternative sexuality

## Abstract

Unconventional sensual, erotic, and sexual behaviors (herein referred to as *kink behaviors*) investigated by academia are based largely on clinical and criminal cases, and most published, peer-reviewed, quantitative research on these behaviors is based almost exclusively on male participants. For this study, information was collected and analyzed from 1580 female participants recruited from the kink community, using a non-clinical and non-criminal sample. We explored and described the preferences and diversity of more than 126 sensual, erotic, and sexual behaviors found among these participants, along with recommendations for continued research. Gaining a better understanding of the breadth and depth of activities engaged in by female kink practitioners could benefit educators, counselors, therapists, medical doctors, and other professionals when interacting with members of the kink community.

## Introduction

Throughout the field of sexology, unconventional sexual behaviors, referred to in this article collectively as *kink behaviors,* have been investigated almost exclusively through clinical and criminal case studies (Ernulf & Innala, 1995; Hugh-Jones, Gogh, & Littlewood, 2005; Weiderman, as cited by Kelly, Bimbi, Nanin, Izienicki, & Parsona, 2009; Richters, Grulich, de Visser, Smith, & Rissel, 2003; Taylor & Ussher, 2001; Weinberg, Williams, & Calhan, 1995). In 1886, Richard von Krafft-Ebing published details of more than 200 clinical cases of unconventional sexual behaviors in the encyclopedia of sexual perversions, *Psychopathia Sexualis:* *With Especial Reference To The Antipathic Sexual Instinct, A Medico*-*Forensic Study* (Ridlinger, 2006; Weinberg, Williams, & Moser, 1984). This influential book was written at a time and place where society and scientists believed that the only purpose of sex was for reproduction; normal healthy sexuality was therefore defined as penile/vaginal intercourse (coitus). As stated by von Krafft-Ebing (1906), “with opportunity for the natural satisfaction of the sexual instinct, every expression of it that does not correspond with the purpose of nature—i.e., propagation—must be regarded as perverse” (p. 79). This fundamentally pathological bias in the scientific literature has led to even mild forms of sexual variations being associated with mental illness or criminality (Ernulf & Innala, 1995).

Almost all of these clinical case studies were based on male clients. von Krafft-Ebing (1906) documented only two cases of female sadism and two cases of female masochism, and acknowledged that he had “thus far not succeeded in obtaining facts with regard to pathological fetishism in women” (p. 24). Early sexologists, such as von Krafft-Ebing and Freud, contended that masochism was the natural state for women; therefore, it was impossible to study female sexual masochism (von Krafft-Ebing, 1906). They postulated that sadism and fetishism were exaggerations of male desires and thus rare in women (Freud, 2000; Kinsey, Pomeroy, Martin, & Gebhard, 1953; von Krafft-Ebing, 1906).

By the 1970s, organizations based on erotic preferences (such as sadomasochism) were sufficiently developed for sex researchers to acknowledge their existence and to begin to study them (Queen, 1996). It has since been established in scientific literature that community-based empirical studies outside of clinical and criminal contexts are necessary. Specifically, sex researchers sought to investigate the behaviors performed by members of the “kink” community (Breslow, Evans, & Langley, 1986; Ernulf & Innala, 1995; Moser & Kleinplatz, 2006; Weinberg et al., 1984).

However, researchers of these subsequent community-based quantitative studies had difficulty obtaining female participants (Moser & Levitt, 1987; Sandnabba, Santtila, & Nordling, 1999). In two quantitative studies that analyzed behaviors of female kink practitioners, the small sample sizes of women were reduced further by researchers omitting responses from professionals (Breslow, Evans, & Langley, 1985; Levitt, Moser, & Jamison, 1994). For example, in the Breslow et al. (1985) study, the researchers received responses from 52 women. The analysis removed 12 of these women’s responses because they had been paid to participate in kink behaviors. Thus, a total of 40 women were examined. In order to compare their data with the Breslow study, researchers Levitt et al. (1994) removed from the analysis any female who was assumed to be an SM prostitute (determined by those having a significantly higher number of SM partners and higher income levels). From their original subsample of 47 women, 2 surveys were illegible and 11 were assumed by the researchers to be SM prostitutes. Thus, the subsample for analysis consisted of 34 non-prostitute, sadomasochistically-oriented women (Levitt et al. 1994).

In part due to these small sample sizes, it was conjectured that few women participated in kink and those who did participate did so only at the request of a male partner or for financial gain (Breslow et al., 1985; Spengler, as cited by Moser & Levitt, 1987). Kink was believed to be a male-only phenomenon with little to no intrinsic interest for women. These assumptions do not leave room for the idea that women enjoy these activities for their own sensual or erotic pleasure nor does it explain the existence of women-only kink events or organizations.

Despite the challenges of recruiting female participants for their study, Moser and Levitt (1987) acknowledged that women were indeed involved in kink behaviors as noted by the “evidence of a large number of women in the S/M subculture” (p. 332). Other researchers have also recognized that enough women participate in the kink community to study (Breslow et al., 1985; Levitt et al., 1994; Moser & Levitt, 1987; Tomassilli, Golub, Bimbi, & Parsons, 2009; Weinberg et al., 1984) and that there is a need for further research in this area. Yet, the academic literature is still lacking. Much of the scientific data regarding females from the kink community are based on a handful of anecdotes, and few of the qualitative studies delve into the variety of their erotic behaviors.

This present research included only female participants in a community-based, non-clinical, non-criminal sample, exploring and describing their preferences and diversity of sensual, erotic, and sexual behaviors. The aim of this study was to build upon the foundations of the previous quantitative research and to expand the scope of knowledge regarding women. Furthermore, this study included behaviors that are likely to be unique to women and therefore have not been scientifically investigated in previous studies.

Research has established the necessity of learning to talk intelligently to kink practitioners (Breslow et al., 1986; Ernulf & Innala, 1995; Moser & Kleinplatz, 2006; Weinberg et al., 1984) and of understanding the language used by them in describing the hundreds of behaviors, relationships, and frameworks found in their community. These considerations may positively impact the effectiveness of educators, counselors, therapists, medical doctors, and other professionals when interacting with members of the kink community (Barker, Iantaffi, & Gupta, 2007; Nichols, 2006). More specifically, further insight into the variety of human sexual expressions may be provided by understanding what women from this community enjoy.

### Definitions

For the purpose of this study, a *kink behavior* refers to unconventional sensual, erotic, and sexual behavior including BDSM-related behaviors (physical and psychological stimuli including bondage, discipline, dominance, submission, sadism and masochism), exhibitionistic behaviors (arousal by being observed by others), voyeuristic behaviors (arousal by observing others), fetishistic behaviors (arousal by objects), and others. Many of these behaviors may be described in clinical research as paraphilias (Fedoroff, Fishell, & Fedoroff, 1999). However, some of these terms used by researchers and by the participants associated with those labels do not necessarily describe the behaviors or attitudes of the participants. Although clinical terms such as *sadism*, *masochism*, and *sadomasochism* have been appropriated by certain groups, members of these groups may use the terms non-clinically. There is no authoritative definition of the non-clinical use of these terms nor is there a consensus regarding the behaviors these labels encompass.

In this paper, the term *kink practitioner* refers to a person who participates in at least one of the kink behaviors described, and the term *kink community* refers to organizations representing kink practitioners. These organizations provide physical or online spaces for adults with similar interests to meet, discuss, support, educate, and/or interact and may also be known as BDSM, Fetish, Leather, Sadomasochistic, S&M, SM, S/M, Alt Sex, and Alternative Sexuality, among others. There may be nuances and differences between these various organizations; however, these differences are outside the scope of this research.

## Method

### Participants

The present study investigated the sensual, erotic, and sexual behaviors of women from the kink community in order to both expand on previous quantitative research found in the literature and address the dearth of data regarding women from this population. A total of 1764 participants began the survey between June 2010 and May 2011. However, only participants who answered at least one question about their sensual, erotic, or sexual behaviors were analyzed. Therefore, 1580 participants (N = 1580) were included in the present analysis. All participants indicated that they were female and over the age of 18. The median age was 34 years, and the mean age was 35.53 with a SD of 10.74. The age range was between 19 and 72 years.

This was an international sample, with nearly 80 % of the participants from the U.S. (women from 49 states responded, with the highest percentage from California), and about 20 % from the following 20 countries: Australia, Austria, Belgium, Brazil, Canada, Czech Republic, Ecuador, England, Finland, France, Germany, India, Ireland, Japan, Mexico, Netherlands, New Zealand, Scotland, Switzerland, and Wales.

About half of the women in the sample (55.31 %) described their relationship status as partnered: either married, in a domestic partnership, or in a long-term relationship. Approximately 42 % were single, divorced/separated, or widowed. A majority of participants (74.33 %) indicated a relationship status not typically asked in demographic surveys. Table 1 provides detailed information on the relationship status of the participants.

**Table 1:** Relationship status of participants as a percentage of the subsample (n = 1383)

Relationship	Percent
Polyamorous/open relationship/polyfidelity	39.91
Married/domestic partner	31.09
Casual BDSM relationship(s)/play partner(s)	30.51
Long-term relationship	30.15
Single (never married/domestic partnership)	21.76
24/7 BDSM relationship	20.03
Divorced/separated	19.38
Monogamous	14.75
BDSM family	11.35
Swingers	4.63
Widowed	1.81
Other	10.20

Since more than one category could be chosen, percent adds up to more than 100

### Measure

For the purpose of this study, questions about these activities were categorized as follows: 62 BDSM-related behaviors, 10 role-play scenarios, 5 forms of exhibitionistic behaviors, 8 forms of erotica, 5 broad categories of fetishistic behaviors, 24 overt sexual activities, and 12 miscellaneous erotic activities. These categories are not rigid and should not be considered as such. Participants provided 72 additional “other” activities through fill-in answers. For each question, the “other” responses were counted only if the information was not simply a clarification of answers.

The survey instrument^1^ was designed to provide data comparable to previous quantitative studies that investigated behaviors of kink practitioners while concurrently investigating a broader spectrum of human sexual behaviors. Participants were asked to indicate which of 126 activities they willingly participated in for their own sensual or erotic pleasure. The survey provided opportunities to add additional comments, including six questions prompting clarification or expansion of answers (fill-in text) and 15 “other” text boxes. Due to practical limitations, only the questions from the survey about demographic information and quantitative information pertaining to behaviors are reported in this article.

### Procedure

The Internal Review Board of San Francisco State University approved this project. Participants were recruited through a variety of kink community events, including social functions, educational workshops, play parties, weekend retreats, and via online forums. Administrators of each group listed on www.thebdsmeventspage.com (that were not specifically male-oriented and that had active websites) were contacted via e-mail and were informed about the nature of the study along with a request to recruit participants. Upon permission from the organizers, recruiting flyers and recruiting scripts were distributed at the event and/or posted online. These materials included the URL link for the online survey.

The first recruitment effort was in June 2010 at a women-only kink community event. The remaining active recruitment efforts took place from August through December 2010. Approval was received from a total of 21 kink organizations during the active recruitment period. Between January and May 2011 (after the recruitment efforts ended and no additional flyers or invitations were distributed), an additional 219 surveys were completed online. Due to the nature of online social networking, some snowball sampling may have occurred (through word-of-mouth, e-mail, and online posts).

### Data Analysis

Answers from the survey were analyzed quantitatively using single variable descriptive statistics and frequency analysis. The following data were collected and analyzed: the activity participated in, the type of participation (doing to, having done to, observing), the total number of activities per person, and the average and range of number of activities participated in.

## Results

The participants of this survey indicated that they participated in a much wider variety of activities than has been acknowledged in academic research of kink behaviors. Out of the 126 erotic stimuli provided in this survey, the women in this sample willingly participated in an average of 57.72 activities with a SD of 22.47. The number of activities participants engaged in ranged from 1 to 121. This does not include the additional 72 categories created by the “other” fill-in responses.

### BDSM-Related Behaviors: Physical and Psychological Stimuli

Table 2 shows the percentage of the women who indicated that they participated in the 62 BDSM-related behaviors provided in the survey. The column “Doing to and/or Done to you” indicates the percentage of the women who participated in the activity in either role whereas the column “Participated (in any form)” also includes responses from “Observing Activity.”

**Table 2:** BDSM behaviors as a percentage of the sample (N = 1580)

Activity	Doing activity to others	Activity done to you	Doing to and/or done to you	Observing activity	Participate (in any form)
Touching (caress, cuddle, massage, tickle)	97.41	97.53	99.37	65.38	99.62
Kissing, licking, sucking	97.09	97.41	99.11	65.32	99.56
Spanking	56.52	85.13	93.99	56.71	95.70
Hair pulling	54.18	84.24	91.33	48.61	93.16
Biting	75.25	81.52	89.75	45.00	92.03
Scratching/leaving marks/abrasion	69.18	74.56	87.85	46.33	90.06
Use bondage toys (chains, gags, cuffs, rope, etc.)	50.82	77.78	85.63	56.90	87.53
Moderate bondage (can’t get out on own, but with body mobility)	42.47	74.05	82.91	55.00	86.39
Light bondage (able to get out if you wanted to)	48.99	76.58	82.53	52.78	85.13
Paddling	43.29	65.32	76.90	53.48	84.24
Breast play: slap, clothespins, etc	34.94	70.19	78.35	50.00	83.16
Flogging	42.72	67.15	75.95	54.62	81.90
Grooming (shaving, manicure, pedicure, brush hair, etc.)	52.85	70.32	77.28	32.97	80.63
Genital play: slap, kick, clothespins, etc	38.16	61.01	73.42	48.48	80.38
Ice play	45.70	63.54	74.49	35.51	80.06
Hickeys	61.65	69.43	76.33	30.13	79.62
Using clothespins/clamps	37.53	62.47	72.97	47.78	78.80
Candle wax play	37.66	57.47	66.84	47.28	78.61
Physical humiliation (face-slapping, begging, crawling, etc.)	32.03	54.43	66.96	52.91	77.53
Pinching	44.81	61.71	73.23	37.66	76.71
Applying ____ (body paint, chocolate, oil, food, etc.)	55.06	62.91	68.99	39.30	76.58
Whipping	28.61	50.25	60.00	52.47	75.70
Deprivation (forced chastity, blindfold, bathroom use control, orgasm control, sensory deprivation, etc.)	31.96	52.15	64.75	41.65	72.03
Stringent/extreme bondage (immobilized)	24.68	46.46	55.00	51.77	70.70
Punishment: physical	26.14	49.43	60.82	41.14	70.32
Obedience/training	23.80	44.81	57.15	41.65	69.56
Using feathers/fur	45.06	54.94	61.96	34.87	68.80
Caning	27.34	45.19	54.68	49.49	68.67
Breath play, choking, strangling, hanging	23.61	51.52	57.59	37.91	66.84
Verbal abuse/humiliation (yelling, calling names, etc.)	25.70	42.72	53.23	44.81	66.77
Electrical play (Violet Wand, TENS unit, etc.)	22.28	40.06	46.84	47.59	63.86
Wrestling	42.28	44.62	47.03	41.52	63.23
Service-oriented submission/domestic service	24.18	37.22	51.39	36.52	61.84
Other forced activities (eating, exercise, masturbation, sexual behavior, nudity, etc.)	22.85	40.76	51.58	35.57	60.38
Knife play/razors	19.30	38.61	44.62	39.24	56.52
Impact play/percussion play: using blunt or heavy instrument	25.06	38.67	46.71	37.85	54.75
Objectification: use person as an object (human furniture, human doll, human ashtray, etc.)	19.37	24.30	36.52	40.38	54.56
Fire play/fire cupping	13.67	26.39	31.08	43.23	53.99
Corset training	8.92	29.56	32.28	34.49	51.52
Piercing: temporary, needles, pins, injections	15.95	26.46	32.03	40.00	50.70
Breast torture (piercings, breast press, stretching, hooks, etc.)	12.72	27.28	32.41	36.14	49.81
Punishment: emotional	15.51	28.92	38.10	29.68	49.62
Attaching weights to body parts	15.13	20.25	29.87	35.13	47.72
Urine play (golden showers/water sports)	19.75	27.15	36.52	26.08	45.70
Piercing: permanent	7.72	29.24	31.65	30.25	44.94
Genital torture (piercing, stretching, stitching, hooks, dilation, etc.)	13.42	16.27	24.62	35.13	44.37
Tattooing: permanent	6.20	29.49	31.14	28.54	43.73
Boxing/beating/kicking	16.08	18.16	24.24	33.23	41.65
Cutting/artistic cutting/scarification	8.67	14.75	18.73	34.37	41.01
Branding/burns	6.39	9.30	13.23	31.08	37.15
Blood play	11.46	16.71	20.51	29.68	36.58
Forced cross-dressing	14.87	2.41	16.14	28.42	35.51
Other torture	13.23	17.47	23.61	25.89	34.37
Vacuum pumping	7.91	15.89	18.99	25.82	34.30
Anal torture	10.82	12.97	19.94	24.49	32.85
Imposed feminization	11.84	3.29	13.86	22.41	28.67
Tattooing: temporary, cell-popping, red-lining	4.75	9.05	11.39	23.92	28.16
Foot torture	8.73	10.51	16.20	20.32	27.53
Water torture	3.80	6.14	8.86	19.87	23.73
Catheterizing	3.67	3.61	6.52	18.54	21.52
Imposed masculinization	3.29	2.15	4.37	13.80	15.32
Feces play (brown showers/scat/excrement/enemas)	3.35	3.99	6.27	10.57	13.73

Other = 2.03 %

The majority of participants enjoyed many kinds of tactile sensations, such as touching (caress, cuddle, massage, tickle), kissing/licking/sucking, grooming, applying (body paint, chocolate, oil, food, etc.), ice play, candle wax play, and using feathers/fur. The two activities that had the highest response rate for erotic and sensual pleasure from the entire survey were touching and kissing. As evidenced by these results, the category of “sensation play without the necessity of pain” is a common phenomenon found among these participants.

It has been noted in previous community-based research that behaviors that may cause pain but are relatively safe tended to be more common than more extreme or dangerous behaviors (described by Moser and Levitt as “more likely to cause medical problems”) (Breslow et al., 1985; Moser & Levitt, 1987). This was consistent with the present findings. Some of the most common among these activities included spanking, hair pulling, biting, and scratching/leaving marks/abrasion. More than half (and in some cases nearly all) of the participants reported that they participated in the following activities, which could be described as “sadomasochism”: breast play (slap, clothespins, etc.), paddling, hickeys, flogging, genital play (slap, kick, clothespins, etc.), pinching, using clothespins/clamps, whipping, and caning.

Some of the more extreme activities (i.e., those with a greater potential to elicit pain or cause physical or psychological damage), while participated in by fewer people, tended to have a higher proportion of women observing these activities for sensual or erotic pleasure. It is possible that more dangerous activities have higher erotic value for the sake of fantasy as opposed to physically experiencing them (i.e., perhaps people do not want to risk harm to themselves or their partners, but are still aroused by fantasized danger). Another possible explanation is that these activities are performed by fewer people because they take a certain amount of training, skill, experience or equipment rather than because of a lack of interest.

The present survey explored psychological components of BDSM behaviors and included 12 activities that could fit this description. The majority of the women from this sample indicated that they participated in at least one of the following activities for their own sensual or erotic pleasure: physical humiliation, deprivation, punishment (physical), breath play, obedience/training, verbal abuse/humiliation, other forced activities, and service-oriented submission/domestic service.

The psychological component of kink is also demonstrated in part by the significant number of women from this sample (n = 1384; 87.59 %) who participated in at least one of the 10 role-play scenarios presented (in any form). As shown in Table 3, the three most popular role-play scenarios were Master/slave fantasy,^2^ danger fantasy, and jobs/occupation play. The columns with “in either role” indicate the percentage of women from this subsample who participated in the role play scenario regardless of which role they took whereas the columns with “Participated (in any form)” combined answers from any of their roles and from observing.

**Table 3:** Role-play scenarios as a percentage of the subsample (n = 1384)

	As the “Top”	As the “bottom”	Top and/or bottom	Observing	Participate (in any form)
Master/slave fantasy play	30.56	60.12	71.24	49.64	83.53
Danger fantasy play	18.06	50.94	58.67	40.82	72.04
Jobs/occupation play (e.g., boss)	27.67	46.24	55.64	40.68	69.44
Animal play	15.10	25.65	34.39	48.27	61.56
Medical play	19.80	23.27	33.31	38.37	53.90
Age regression play (e.g., guardian/child)	14.60	29.62	36.05	35.40	53.83
Religious play (e.g., priest/nun)	8.16	11.85	15.75	24.93	32.30
Incest play (fantasy)	8.45	19.94	22.76	16.18	29.62
Age progression play	0.79	0.87	1.45	8.24	9.39
Sex with corpse (fantasy)	0.94	3.18	3.61	5.42	7.80

Other = 4.77 %. Terms for “Top” category included “Master,” “someone in charge,” “trainer,” “caretaker.” Terms for “bottom” category included “slave,” “subordinate,” “animal,” and “someone being taken care of”

The present results support the idea that bondage is one of the more preferred activities. In fact, more than 85 % of the participants indicated that they have used bondage toys for sensual or erotic pleasure. There was almost no difference between the number of participants of light bondage and moderate bondage (each at over 80 %), which were defined in this survey as “Able to get out if you wanted to” and “Can’t get out on own, but with body mobility,” respectively. More than half of the participants engaged in stringent/extreme bondage (immobilized).

### Exhibitionistic and Voyeuristic Behaviors

More than half of the survey sample (n = 887; 56.14 %) indicated that they participated in at least one of the five categories of exhibitionistic behavior (erotic pleasure from being observed) listed in the survey. As shown in Table 4, the majority of this subsample enjoyed showing bare breasts, followed by engaging in public sex, being naked, acting out sexual fantasy/role play, and showing genitals. More than one half of the participants indicated that they posed for erotic images and/or that they shared erotic images of themselves with others for their own sensual or erotic pleasure (see Table 5 for details). As shown in Table 6, most of the participants (82.78 %) indicated that they created or observed at least one of the following forms of erotica for their own sensual or erotic pleasure: erotic literature, sexual pornography, and observing performances and nude paintings, among others.

**Table 4:** Exhibitionistic behaviors as a percentage of the subsample (n = 887)

Activity	Percent
Showing bare breasts	71.36
Engaging in public sex	57.27
Being naked	50.17
Acting out sexual fantasy/role play	46.67
Showing genitals	37.09
Other	11.61

**Table 5:** Forms of erotica via images as a percentage of the subsample (n = 1101)

Category	Percent
Pose for erotic images (photography, video, audio)	87.83
Share erotic images of yourself with others	78.56
Take erotic images of others (photography, video, audio)	46.32
Share erotic images of your partner with others	28.70
Other	5.45

**Table 6:** Additional forms of erotica as a percentage of the subsample (n = 1308)

Category	Percent
Erotic literature (books, poems, magazines)	90.90
Sexual pornography	74.01
Performance (strip-tease, burlesque, belly dance)	53.13
Nude paintings, sculptures, or statues	35.70
Other	3.90

Many women who responded to this survey reported voyeuristic tendencies from observing others (in a non-clinical sense). When asked if they received sensual or erotic pleasure by observing any of the 96 activities, 1366 (86.46 %) of the 1580 surveyed responded affirmatively, with each of the 96 activities being observed at least once. Receiving pleasure by observing may be a more common phenomenon among women than previously considered.

### Fetishistic Behaviors

A total of 1192 (75.44 %) women indicated that they have been sexually aroused by an object in at least one of the five categories of objects presented in the survey. Clothing, specific body part(s), and fabrics were the most popular categories of this subsample, as shown in Table 7. This study does not address the degree of preference or fixation a participant may have with a particular fetish or fetishes, but rather, takes a non-clinical approach to arousal by objects not specifically designed for sexual response.

**Table 7:** Fetishistic behaviors as a percentage of the subsample (n = 1192)

Category	Percent
Clothing (lingerie, shoes, corsets, etc.)	75.92
Specific body part(s)	61.91
Fabrics (leather, rubber, vinyl, etc.)	56.54
Uniforms (military, medical, occupational, etc.)	51.85
Body fluids	26.43
Other	12.33

### Overt Sexual Behaviors

Researchers have examined additional erotic activities that are not necessarily defined as BDSM behaviors. Specifically, oral sex, anal sex, rimming, and masturbation have been mentioned in previous studies, and, with the exception of rimming, all have been found to be common among male and female participants (female participants were not asked about rimming in these previous studies) (Breslow et al., 1985; Levitt et al., 1994; Moser & Levitt, 1987; Sandnabba et al., 1999). Participants in this study were queried regarding 24 overt sexual activities and were offered a choice of up to 5 categories for each sexual activity. These categories included “Doing Activity to Women,” “Doing Activity to Men,” “Having Activity Done by Women,” “Having Activity Done by Men,” and “Observing Activity.” In Table 8, the column “Doing, having done, and/or both” indicates the percentage of women from this subsample who participated in the activity in any role whereas the column “Participated (in any form)” also includes responses from “Observe Activity.”

**Table 8:** Overt sexual activities as a percentage of the subsample (n = 1394)

Activity	Doing activity to women	Doing activity to men	Having activity done by women	Having activity done by men	Doing, having done, and/or both	Observing activity	Participate (in any form)
Cunnilingus (stimulating a woman’s genitals with mouth)	62.48	8.82	62.55	83.14	95.41	59.54	97.06
Hand job (stimulating genitals with hands/fingers)	47.63	83.93	47.85	59.61	93.19	46.99	93.83
Fellatio (stimulating a man’s genitals with mouth)	3.30	83.57	10.98	13.92	89.45	50.22	91.89
Stimulating anus with fingers or penis	33.93	59.90	35.44	73.17	89.38	46.13	91.39
Penetrating vagina with penis	6.89	17.00	7.75	78.34	86.30	47.20	89.74
Stimulating vagina with other sex toys (vibrators, Ben Wa Balls, speculums, etc.)	51.58	5.31	51.08	67.65	86.73	49.21	88.95
Genital to genital contact: non-penetrative	44.84	64.85	43.40	62.12	81.06	37.45	83.00
Ingesting semen	3.01	65.85	8.54	14.56	75.39	36.30	79.12
Stimulating anus with sex toys (beads, butt plug, vibrator, speculum, hooks, etc.)	23.10	41.03	25.39	54.09	74.53	38.38	78.19
Mammary intercourse (rubbing phallic object or penis between breasts)	10.26	15.28	10.11	59.18	71.74	34.22	76.69
Rimming (stimulating anus with mouth)	18.87	40.32	21.52	49.14	64.20	34.07	70.30
Worship (kissing, licking, smelling, enjoying a specific body part)	28.62	47.42	28.55	43.40	66.28	32.64	69.37
Stimulating penis with sex toys (cock rings, sheath, etc.	2.80	50.50	7.17	13.63	56.03	39.89	65.64
Using a strap-on dildo to penetrate vagina	35.80	4.81	36.37	13.49	49.21	44.55	64.35
Sucking/licking dildo with mouth	28.55	17.43	30.06	27.12	53.95	40.17	63.99
Ingesting vaginal fluid	40.32	5.09	38.59	44.91	59.97	34.72	63.56
Vaginal fisting	24.18	1.43	22.02	30.99	49.07	38.67	62.55
Using a strap-on dildo to penetrate anus	16.79	33.07	16.86	10.83	44.98	36.08	57.39
Foot job (stimulating genitals with feet)	10.04	33.57	8.25	13.99	41.75	24.03	49.35
Anal fisting	4.09	12.34	4.38	4.73	17.50	25.47	34.86
Passing female vaginal fluid from mouth-to-mouth	16.36	6.24	15.49	15.49	23.31	18.01	30.13
Snowballing (passing semen from mouth-to-mouth)	6.17	14.78	5.52	7.60	19.66	20.30	30.13
Felching (licking semen out of anus)	1.72	1.58	1.79	3.08	4.30	11.12	12.70
Using semen or vaginal fluid in mixed-drinks or cooking/baking recipes	2.01	3.95	1.65	3.52	6.31	5.67	9.54

Other = 3.37 %

Based on the 1394 participants who responded to the question about overt sexual activities, the present results found that oral sex (cunnilingus and fellatio) and anal sex were among the more common of these activities. In fact, as shown in Table 8, each of the following overt sexual activities was more common than coital sex (penetrating vagina with penis) from this subsample: oral sex, including cunnilingus and fellatio, hand job (stimulating genitals with hands/fingers), and anal sex (stimulating anus with fingers or penis).

Other popular overt sexual activities (between 40 and 88 %) included stimulating vagina with other sex toys, genital-to-genital contact: non-penetrative, ingesting semen, stimulating anus with sex toys, mammary intercourse (rubbing phallic object or penis between breasts), worship (kissing, licking, smelling, enjoying a specific body part), rimming (stimulating anus with mouth), ingesting vaginal fluid, stimulating penis with sex toys (cock rings, sheath, etc.), sucking/licking dildo with mouth, using a strap-on dildo to penetrate vagina, vaginal fisting, using a strap-on dildo to penetrate anus, and foot job (stimulating genitals with feet).

### Miscellaneous Erotic Behaviors

Almost all of the 1376 participants who answered the question about additional erotic activities (see Table 9 for details) indicated that they masturbated (solo). Solo masturbation rates were higher in this study (at 86.01 % of the entire sample) as compared to female participants from previous studies, including the Kinsey et al. (1953) study at 62 %, 73 % from the Breslow et al. (1985) study, and 59 % from the Levitt et al. (1994) study. It is difficult to ascertain why this disparity exists. Possibilities include the notion that these women were more likely to be comfortable enough with their sexuality to engage in this behavior, that the taboo of masturbation is less prevalent now than in the past, that these women were more comfortable admitting to this behavior, or that masturbation is more prevalent for women from this particular population. Mutual masturbation (with a partner) and phone sex were also popular activities, followed by sex with inanimate objects, cyber sex (webcam), group sex, anonymous sex/sex with strangers, and swinging.

**Table 9:** Miscellaneous erotic activities as a percentage of the subsample (n = 1376)

Activity	Percent
Masturbation (solo)	98.76
Mutual masturbation (with a partner)	90.26
Phone sex	71.88
Sex with inanimate objects (not designed as sex toys)	56.03
Cyber sex (webcam)	50.80
Group sex (including orgies, gang bang, bukkake)	45.71
Anonymous sex/sex with strangers	34.74
Swinging (mate swapping)	29.58
Prostitution (real)	6.83
Sex with domestic animals (real)	5.45
Sex with wild/non-domestic animals (real)	0.36
Sex with a dead person (real)	0.07
Other	4.29

### Female-Specific Erotic Behaviors

The survey included activities that are likely to be unique to females (and therefore had not been addressed in previous quantitative studies about kink behaviors). These activities included hair pulling, breast play, corset training, breast torture, imposed masculinization, showing bare breasts, ingesting vaginal fluid, passing female vaginal fluid from mouth-to-mouth, using a strap-on dildo to penetrate vagina, stimulating vagina with other sex toys, vaginal fisting, mammary intercourse, using a strap-on dildo to penetrate anus, and non-penetrative genital to genital contact. Additional categories from the fill-in responses that also may be unique to women included lactation play (such as adult breastfeeding/adult erotic nursing/drinking or squirting of breast milk), stimulating vagina with breast, blood play using menstrual blood, cunnilingus on menstruating woman, stimulation of g-spot, clitoris, or nipples, double/triple/multiple penetration (vaginal, anal, oral), cuckolding, using one partner to have sexual encounters with another person, squirting vaginal fluid, swallowing and playing with own vaginal fluids, and strap-on cock sucking. Very little data about these activities exist in the scientific literature.

### Combined Results

Table 10 shows the percentage of the entire sample that participated in an activity (in any form). When considering all forms of sensual, erotic, and sexual behaviors listed in the survey, the 20 most common activities (in any form) were as follows (in order from most common to least common): touching, kissing/licking/sucking, spanking, hair pulling, biting, scratching/leaving marks/abrasion, use bondage toys, moderate bondage, masturbation (solo), cunnilingus, light bondage, paddling, breast play, hand job, flogging, fellatio, grooming, stimulating anus with fingers or penis, genital play, and ice play. This was somewhat similar to previous research. From their female sample, Levitt et al. (1994) reported “clear preferences for bondage, spanking (the traditional bondage and discipline duo), oral sex, and the master–slave game…” (p. 471). However, in this present sample there were several other activities that were equally as common, or more common, including light sensations/tactile play, masturbation, breast play, and anal play.

**Table 10:** Sensual, erotic, and sexual behaviors participated in (in any form) as a percentage of the entire sample (*n* = 1580)

Activity	Percent
Touching (caress, cuddle, massage, tickle)	99.62
Kissing, licking, sucking	99.56
Spanking	95.70
Hair pulling	93.16
Biting	92.03
Scratching/leaving marks/abrasion	90.06
Use bondage toys (chains, gags, cuffs, rope, etc.)	87.53
Moderate bondage (can’t get out on own, but with body mobility)	86.39
Masturbation (solo)	86.01
Cunnilingus (stimulating a woman’s genitals with mouth)	85.63
Light bondage (able to get out if you wanted to)	85.13
Paddling	84.24
Breast play: slap, clothespins, etc.	83.16
Hand job (stimulating genitals with hands/fingers)	82.78
Flogging	81.90
Fellatio (stimulating a man’s genitals with mouth)	81.08
Grooming (shaving, manicure, pedicure, brush hair, etc.)	80.63
Stimulating anus with fingers or penis	80.63
Genital play: slap, kick, clothespins, etc.	80.38
Ice play	80.06
Hickeys	79.62
Penetrating vagina with penis	79.18
Using clothespins/clamps	78.80
Candle wax play	78.61
Sex with inanimate objects (not designed as sex toys)	78.61
Stimulating vagina with other sex toys (vibrators, Ben Wa Balls, speculums, etc.)	78.48
Physical humiliation (face-slapping, begging, crawling, etc.)	77.53
Pinching	76.71
Applying ____ (body paint, chocolate, oil, food, etc.)	76.58
Whipping	75.70
Erotic literature (books, poems, magazines)	75.25
Genital to genital contact: non-penetrative	73.23
Master/slave fantasy	73.16
Deprivation (forced chastity, blindfold, bathroom use control, orgasm control, sensory deprivation, etc.)	72.03
Stringent/extreme bondage (immobilized)	70.70
Punishment: physical	70.32
Ingesting semen	69.81
Obedience/training	69.56
Stimulating anus with sex toys (beads, butt plug, vibrator, speculum, hooks, etc.)	68.99
Using feathers/fur	68.80
Caning	68.67
Mammary intercourse (rubbing phallic object or penis between breasts)	67.66
Breath play, choking, strangling, hanging	66.84
Verbal abuse/humiliation (yelling, calling names, etc.)	66.77
Electrical play (Violet Wand, TENS unit, etc.)	63.86
Wrestling	63.23
Danger fantasy (abduction, execution, impregnation, interrogation, rape, kidnapping, prison scene)	63.10
Phone sex	62.59
Rimming (stimulating anus with mouth)	62.03
Service-oriented submission/domestic service	61.84
Sexual pornography	61.27
Pose for erotic images (photography, video, audio)	61.20
Worship (kissing, licking, smelling, enjoying a specific body part)	61.20
Jobs/occupation play (boss/secretary, maid, teacher/student)	60.82
Other forced activities (eating, exercise, masturbation, sexual behavior, nudity, etc.)	60.38
Stimulating penis with sex toys (cock rings, sheath, etc.)	57.91
Clothing (lingerie, shoes, corsets, etc.)	57.28
Using a strap-on dildo to penetrate vagina	56.77
Knife play/razors	56.52
Sucking/licking dildo with mouth	56.46
Ingesting vaginal fluid	56.08
Vaginal fisting	55.19
Impact play/percussion play: using blunt or heavy instrument	54.75
Share erotic images of yourself with others	54.75
Objectification: use person as an object (human furniture, human doll, human ashtray, etc.)	54.56
Fire play/fire cupping	53.99
Animal play (pony, kitten, wolf, etc.)	53.92
Corset training	51.52
Piercing: temporary, needles, pins, injections	50.70
Using a strap-on dildo to penetrate anus	50.63
Breast torture (piercings, breast press, stretching, hooks, etc.)	49.81
Punishment: emotional	49.62
Sex with inanimate objects (not designed as sex toys)	48.80
Attaching weights to body parts	47.72
Medical play (doctor or nurse/patient)	47.22
Age regression play: guardian/child (baby, toddler, teenager, brat, etc.)	47.15
Specific body part(s)	46.71
Urine play (golden showers/water sports)	45.70
Piercing: permanent	44.94
Genital torture (piercing, stretching, stitching, hooks, dilation, etc.)	44.37
Cyber sex (webcam)	44.24
Performance (strip-tease, burlesque, belly dance)	43.99
Tattooing: permanent	43.73
Foot job (stimulating genitals with feet)	43.54
Fabrics (leather, rubber, vinyl, etc.)	42.66
Boxing/beating/kicking	41.65
Cutting/artistic cutting/scarification	41.01
Showing bare breasts	40.06
Group sex (including orgies, gang bang, bukkake)	39.81
Uniforms (military, medical, occupational, etc.)	39.11
Branding/burns	37.15
Blood play	36.58
Forced cross-dressing	35.51
Other torture	34.37
Vacuum pumping	34.30
Anal torture	32.85
Take erotic images of others (photography, video, audio)	32.28
Engaging in public sex	32.15
Anal fisting	30.76
Anonymous sex/sex with strangers	30.25
Nude paintings, sculptures, or statues	29.56
Imposed feminization	28.67
Religious play (priest/nun, priest/altar boy or altar girl)	28.29
Tattooing: temporary, cell-popping, red-lining	28.16
Being naked	28.16
Foot torture	27.53
Passing female vaginal fluid from mouth-to-mouth	26.58
Snowballing (passing semen from mouth-to-mouth)	26.58
Acting out sexual fantasy/role play	26.20
Incest play (fantasy)	25.95
Swinging (mate swapping)	25.76
Water torture	23.73
Catheterizing	21.52
Showing genitals	20.82
Share erotic images of your partner with others	20.00
Body fluids	19.94
Imposed masculinization	15.32
Feces play (brown showers/scat/excrement/enemas)	13.73
Felching (licking semen out of anus)	11.20
Using semen or vaginal fluid in mixed-drinks or cooking/baking recipes	8.42
Age progression (elderly)	8.23
Sex with corpse (fantasy)	6.84
Prostitution (real)	5.95
Sex with domestic animals (real)	4.75
Sex with wild/non-domestic animals (real)	0.32
Sex with a dead person (real)	0.06

## Discussion

The information gleaned by this research contributes to the body of knowledge regarding kink behaviors, specifically from a female perspective. The present study investigated the sensual, erotic, and sexual behaviors of 1580 women from the kink community in order to both expand on previous quantitative research found in the scientific literature and to address the dearth of data regarding females from this population. The survey included 126 forms of erotic stimuli—a broader spectrum of human sexual behavior than documented in previous studies of kink practitioners. Included in these forms of erotic stimuli were behaviors that may be unique to female participants. These behaviors have not been included in the scientific literature since most of the studies about activities found within the kink community are based on male participants and behaviors.

Previous studies have removed female professional sex workers (or those assumed to be) from their analysis (Breslow et al., 1985; Levitt et al., 1994). Results from this study show that many professionals in the sex industry nevertheless receive sensual or erotic pleasure from their professional activities. Many of the participants who indicated that they had been paid money for erotic services also indicated they enjoyed their work, that their work satisfied their erotic desires, and that they still participated in these activities on a personal level. Some of the professions stated in fill-in responses included the following occupations: fetish model, bondage model, professional dominatrix, professional submissive, sex worker, owner of adult websites, pornography actress, and webcam model. These professional women are part of the kink community and their input should not be discounted. More than 97 % of these professionals indicated that they participate in kink behaviors for at least one reason other than for money.

This study reaffirms the existence of female kink practitioners. The women who participated in this study indicated that they each willingly engaged in kink behaviors for their own enjoyment. The number of women who responded to the survey, the number of activities each woman was involved with, and the range of activities participated in, considered with the willingness to disclose such details, indicate a vibrant, ardent participation in the kink community that has thus far outpaced academic perceptions. The results of the study expand our understanding of the depth and breadth of female sexual expression.

### Limitations

Limitations of this study include the categorization of behaviors, the subjectivity of terms, the inherent limits of online data collection, and sampling methods. Categorization was problematic. Some activities can be grouped into more than one category; for example, “choking on cock” could be categorized as a sadomasochistic act or it could be labeled as fellatio and fall under the category of overt sexual activity. Some participants pointed out that, from their perspective, the behaviors were miscategorized or too general. For example, enema play should have been separated from fecal play, as some participants stated that they engaged in enema play without being in contact with their partner’s feces. Also, the survey asked participants to indicate only the behaviors they participated in willingly for their own sensual or erotic pleasure, yet it is possible that members of the kink community participate in many of the activities listed for nonsexual purposes. For example, some women from this sample mentioned in open-ended questions that they participated in these behaviors for spiritual, medical, or therapeutic purposes. Therefore, it is necessary to reconsider how erotic stimuli and kink behaviors are categorized.

The terms used for the activities are subjective and may have different meanings to different participants. Also, certain terms used in the kink community may have different meanings than they do in the general population. For example, there is a difference between what people from the kink community call “torture” and real torture, and it is understood that “slaves” from the kink community are not being held against their will as contrasted with the sex trade industry or criminal kidnappings. Additionally, the distinction between “doing an activity,” “having an activity done to you,” and “observing an activity” is imprecise and may also be subjectively interpreted by participants. For example, kissing may be seen as doing to, having done to, or both.

A request to participate in this study was distributed by flyers to various events and organizations in the kink community, both at physical locations and online. However, the survey itself was available only online, limiting participation to people with Internet access and sufficient opportunity to complete it. This method also limited the ability of participants to ask for clarification or expansion of ideas or topics. It is possible that behaviors listed in the beginning of the survey had a higher response rate due to survey fatigue or some of the participants dropping out of the study. As with any anonymous survey, one must be cautious since there is no effective method to verify the responses.

This study was based on a community-based, non-random sample. Therefore, results should not be generalized to the general population. Without knowledge of the total number of kink practitioners, the sample size may not be statistically significant for the results to be representative of the entire kink community. It is reasonable to assume that there are many people practicing kink behaviors who have no knowledge of the kink community; it is also reasonable to assume that many people practice kink behaviors but do not label the behaviors as kink or think of themselves as part of the community. It is likely that these people were not included in this study.

### Recommendations for Future Research

This present study was exploratory and descriptive, and it revealed many possible avenues for future research. Further analysis of data from this study and collecting additional data are also recommended.

Breslow et al. (1985) noted that the participants tended to have a lower rate of marriage compared to the general population (more were single or divorced). In the present study, 74.33 % indicated a relationship status not typically asked in demographic surveys (i.e., single, divorced, married, or widowed). Further investigations are warranted to better understand the complexity and variety of relationship dynamics.

The survey asked only if the women had participated in an activity willingly and for their own sensual or erotic pleasure; this does not address the degree of preference for any particular activity. The connections between desires, fantasies, participation, observation, opportunity, and sexual orientation and identity should be examined in more detail, perhaps with a longitudinal study in order to capture changes over time. This study did not differentiate between having participated only once, as an occasional activity, as an integral part of sexual patterns, or as a favorite activity. Furthermore, the reasons for participating in kink activities were addressed only on a general level. More nuanced information about the motivations behind specific behaviors would require further investigation.

The activities provided in the survey did not measure levels of intensity of stimulation. For example, spanking could mean a light tap on the buttocks, a slap that leaves a welt, or an accumulation of light taps that together provide a stinging sensation. Different people may perceive pain and intense stimulation in different ways. One of the study participants described having fibromyalgia and allodynia, and she used kink behaviors as a way of dealing with her chronic pain. This phenomenon warrants further investigation.

The issue of safety and consent were mentioned on several occasions in the fill-in responses, including the acronyms SSC (Safe, Sane, and Consensual) and RACK (Risk Aware Consensual Kink). One possible explanation of the survey results showing the more “dangerous” activities to be engaged in by fewer participants may be due to an emphasis on safety in some parts of the community.

Further analysis of the open-ended questions answered in this survey will likely provide a more detailed understanding of preferences, motivations, relationship dynamics, and various aspects of the kink community. Some of these fill-in responses included favorites (such as erotic activities, objects, role-play scenarios, and erotica themes), and descriptions of experiences, meanings, and lifestyles. Additionally, in order to provide even more insight into the phenomenon of kink and of female sexuality, a more in-depth analysis should be made of the role preferences (i.e., “Doing to” and “Done to you”), the relationship between observing and participating in an activity, and the additional categories provided by fill-in responses.

## Footnotes

1 A copy of the entire questionnaire is available from the author upon request.

2 Some of the study participants commented that Master or Mistress and slave (a.k.a. “M/s”) or Dominant or Domme and submissive (a.k.a. “D/s”) was not merely a role-play scenario for them, but rather, an integral aspect of their relationship and/or their identity.
